# Responsibility of Practitioners—Poisoning by Mistake

**Published:** 1836-01

**Authors:** 


### RESPONSIBILITY OF PRACTITIONERS.—POISONING BY MISTAKE.

An army physician and an apothecary have been fined the sum of two and three hundred francs at Bruges under the following circumstances. The physician had prescribed an emollient enema for one of his patients; but, as it was eleven at night, and the apothecary was in bed, he prepared it himself, with the assistance of one of the servants of the hospital. After having poured into the syringe part of the decoction of senna which he had made, he went to the dispensary, awoke the apothecary, and asked him for some linseed oil: the apothecary took down a bottle from a shelf, and put it on the counter. The physician took it away, and poured part of its contents into the syringe. Unfortunately he neither discovered, from the effervescence produced by the mixture, nor by the vapour, nor smell, (all of which were remarked by the attendant,) that the fluid was sulphuric acid. The clyster was given, and immediately afterwards the patient uttered the most horrible shrieks, and passed the night in acute pain: no remedies were availing, and he died.

*Journal de Chimie Medicale.* Août, 1835.

